# En-route to the ‘elimination’ of genotypic chloroquine resistance in Western and Southern Zambia, 14 years after chloroquine withdrawal

**DOI:** 10.1186/s12936-019-3031-4

**Published:** 2019-12-03

**Authors:** Lungowe Sitali, Mulenga C. Mwenda, John M. Miller, Daniel J. Bridges, Moonga B. Hawela, Elizabeth Chizema-Kawesha, James Chipeta, Bernt Lindtjørn

**Affiliations:** 1 Centre for International Health, Faculty of Medicine, University of Bergen, Bergen, Norway; 2 Department of Biomedical Science, School of Health Sciences, University of Zambia, Lusaka, Zambia; 3 School of Medicine and University Teaching Hospital Malaria Research Unit (SMUTH-MRU), Lusaka, Zambia; 4 PATH Malaria Control and Elimination Partnership in Africa (MACEPA), National Malaria Elimination Centre, Ministry of Health, Chainama Grounds, Lusaka, Zambia; 5 Malaria Elimination Centre, Ministry of Health, Chainama Hospital and College Grounds, Lusaka, Zambia; 6 Department of Paediatrics and Child Health, University of Zambia School of Medicine, Lusaka, Zambia

**Keywords:** *Plasmodium falciparum*, Chloroquine resistance, Chloroquine sensitivity, Genotypes, Zambia

## Abstract

**Background:**

Anti-malarial resistance is, and continues to be a significant challenge in the fight against malaria and a threat to achieving malaria elimination. In Zambia, chloroquine (CQ), a safe, affordable and well-tolerated drug, was removed from use in 2003 due to high levels of resistance evidenced with treatment failure. This study sought to investigate the prevalence of chloroquine resistance markers in Southern and Western Provinces of Zambia 14 years after the withdrawal of CQ.

**Methods:**

Data from a cross-sectional, all-age household survey, conducted during the peak malaria transmission season (April–May 2017) was analysed. During the all-age survey, socio-demographic information and coverage of malaria interventions were collected. Consenting individuals were tested for malaria with a rapid diagnostic test and a spot of blood collected on filter paper to create a dried blood spot (DBS). Photo-induced electronic transfer–polymerase chain reaction (PET–PCR) was used to analyse the DBS for the presence of all four malaria species. *Plasmodium falciparum* positive samples were analysed by high resolution melt (HRM) PCR to detect the presence of genotypic markers of drug resistance in the *P. falciparum* chloroquine resistance transporter (*Pfcrt*) and *P. falciparum* multi-drug resistance (*Pfmdr*) genes.

**Results:**

A total of 181 *P. falciparum* positive samples were examined for *pfcrt* K76T and MDR N86. Of the 181 samples 155 successfully amplified for *Pfcrt* and 145 for *Pfmdr* N86. The overall prevalence of CQ drug-resistant parasites was 1.9% (3/155), with no significant difference between the two provinces. No N86Y/F mutations in the *Pfmdr* gene were observed in any of the sample.

**Conclusion:**

This study reveals the return of CQ sensitive parasites in Southern and Western Provinces of Zambia 14 years after its withdrawal. Surveillance of molecular resistant markers for anti-malarials should be included in the Malaria Elimination Programme so that resistance is monitored country wide.

## Background

The goal of the Zambia National Malaria Elimination Programme is to eliminate malaria by 2021, through effective and sustained coverage of proven vector control interventions, which include indoor residual spraying (IRS), distribution of long-lasting insecticide-treated nets (LLINs) and larval source management (LSM), combined with prompt effective case management, health promotion, surveillance and research [1]. While the malaria map has been shrinking over the years, there are a number of challenges in maintaining control or progressing to elimination, that include: access to treatment for most at risk and hard to reach populations; insecticide resistance; residual or outdoor transmission and drug resistance, specifically the threat of resistance to artemisinin and artemisinin-based combination therapy [2].

Anti-malarial drug resistance is defined as the ability of *Plasmodium* parasite strain to survive and/or multiply despite the administration and absorption of a drug in a dose equal to or higher than the recommended dose, but within tolerance of the human subject [3]. Resistance to chloroquine (CQ) in *P. falciparum* parasites is predominantly linked to a single mutation in the *P. falciparum* transporter gene (*Pfcrt*) on chromosome 7, which encodes a protein localized on the parasite digestive vacuole (DV) membrane. The replacement of lysine (K) at position 76 to a threonine (T), i.e. the K76T mutation, has been established as the most important prognostic marker of treatment failure [4, 5].

CQ acts against the intra-erythrocytic (trophozoite and schizont) stages of the parasite that are responsible for the clinical manifestation of the disease. The intra-erythrocytic stages digest erythrocytic haemoglobin in acidic vacuoles, releasing toxic haem as a by-product which is then biocrystallized into non-toxic haemozoin [6]. CQ interrupts this detoxification process [7], thus poisoning the parasite [8]. CQ resistant (CQR) *P. falciparum* parasites survive by reducing the accumulation of CQ in the food digestive vacuole thus inhibiting haemozoin formation [6, 9].

Another point mutation N86Y in *P. falciparum* multidrug resistance gene 1 (*Pfmdr1*), on chromosome 5, that encodes a P-glycoprotein homologue and is located on the parasite DV membrane has also been implicated in CQ resistance [4, 5]. The exact mechanism by which this is achieved remains unknown, it is likely mediated by an ATP dependent transporter [9].

However, the mutation in *Pfcrt* is required to confer a basic level of resistance before mutations in *Pfmdr1* can have an effect [10].

CQ was introduced in 1940 and by 1950 it had become the most widely used anti-malarial. It was the mainstay for presumptive treatment and mass drug administration during the era of malaria eradication campaigns; while in tropical Africa where there was no systematic campaign it effectively replaced quinine in the 1970s [11, 12]. From the early 1980s, there had been observed decreases in CQ efficacy, and increased number of clinical recrudescence and treatment failures [13–16]. Therapeutic efficacy studies in Zambia [17] and other neighbouring countries [12] also provided evidence for the resistance. This led to Zambia becoming the first African country to abandon CQ and adopt artemether–lumefantrine (AL) as the first-line treatment nationwide in 2002 [18].

Over the years following the withdrawal of CQ, a number of countries, e.g. China in 2017 [19], Malawi in 2014 [20] and parts of Zambia in 2016 [21] have reported the return of CQ susceptibility, potentially raising the prospect of its reintroduction in the future. This study, therefore, aimed to assess the status of CQ resistance marker in Western and Southern Provinces, Zambia to determine whether CQ sensitivity has continued to return.

## Methods

### Study area

Western and Southern Provinces cover areas of approximately 126,000 km^2^ and 86,000 km^2^, respectively, representing ~ 28% of the total Zambia landmass. They are home to approximately 0.9 and (Western) 1.6 (Southern) million people according to the 2010 population census. The Zambezi River flows through both provinces and the plains cover an area of about 10% of the total area of the Western Province. The predominant ethnic groups are the Tonga speaking people in Southern Province and the Lozi speaking people in Western Province [22, 23].

### Study design

This study aimed to assess the levels of CQ resistance in selected parts of Western and Southern Provinces using samples taken from an all age household cross-sectional survey. The survey was conducted in the two Provinces of Zambia during the peak malaria transmission season in April and May 2017. The Ministry of Health, PATH-Malaria Control and Elimination Partnership in Africa (MACEPA), and other partners carried out this survey as part of the ongoing efforts to evaluate malaria elimination efforts. From the survey, a subset of known malaria test RDT positive samples were analysed for *Pfcrt* and *Pfmdr* markers. In the two provinces sampled, malaria transmission varies greatly, with traditionally higher transmission intensity in Southern Province along Lake Kariba and in areas of Western Province around the swamps and wetland in Luampa, Kaoma and Nkeyema districts and along the Zambezi River basin [24–26]. The rest of the areas away from water bodies generally have low transmission.

Selected households were visited by field teams consisting of trained survey data collectors using standardized questionnaires based on the 2015 Malaria Indicator Survey (MIS) questionnaire to collect socio-demographic characteristics of household members and information on coverage of malaria interventions. In addition, malaria testing using malaria rapid diagnostic tests (SD Bioline Pf) was done on household members of all ages and dried blood spots were collected on Whatmann No 3 filter paper. Informed consent/assent was obtained from the participants that were included in the study [26, 27]. Samples were analysed at the National Malaria Elimination Centre (NMEC) Laboratory.

### Sampling

The survey sampling methods in each province were different due to historical studies and enumeration in the two provinces. In Southern Province, sampling of households was done randomly based on a pre-existing sampling frame used during a previously-implemented mass drug administration (MDA) trial from the 10 districts along Lake Kariba. This involved the random selection of 52 households from each of the 60 health facility catchment areas used during the trial [25]. For Western Province, with no pre-existing household sampling frame, a 2-stage cluster sampling method using probability proportional to their size (PPS) was employed. Using standardized methods from the MIS and national census data [25, 26], 25 households were selected from 24 standard enumeration areas. Across both surveys, a total sample size of 6977 people was attained. For this study, after excluding clusters with zero prevalence, 13 clusters were randomly selected and all the1567 (22% of total samples collected in the 13 clusters) samples screened for *Plasmodium* species by PET–PCR. A total of 266/1567 (16.9%) were *P. falciparum* positive. Due to cost of processing, 181 of these positives were randomly selected for the presence of *Pfcrt* K76T and *Pfmdr* N86Y/F.

### Laboratory methods

#### DNA extraction

DNA was extracted either individually or in pools using Qiagen DNA mini kit (Qiagen, Germany) using a 6 mm punch (~ 13.8 µl whole blood) taken from each DBS according to the manufacturer’s instructions with modifications. All RDT positive samples were extracted individually, while RDT negatives were pooled in groups of 10 and the positive pools were deconvoluted.

#### PET–PCR

This was performed as previously described by Lucchi et al. in 2013 [28]. Briefly, 5 µl of DNA template, 2X TaqMan Environmental Master Mix 2.0 (Applied BioSystems, Life Technology LTD, Warrington, UK) and 250 nM each forward and reverse primers were amplified in a 20 µl reaction volume as follows; initial hot-start at 95 °C for 15 min, followed by 45 cycles of denaturation at 95 °C for 20 s, annealing and extension at 60 °C for 40 s. Samples were tested in duplicate and scored positive if both duplicates had a critical threshold (CT) value < 40 [29, 30].

#### Pre-amplification

*Plasmodium falciparum* positive samples were pre-amplified using a Pre-amplification master mix (Life Technologies, Inc, Grand Island, NY, USA) and a mixture of the pooled primers in a 10 µl (CT > 35) or 20 µl (CT < 35) reaction volume to enhance the template concentration. The following amplification conditions were used: pre-incubation at 95 °C for 2 min, amplification at 95 °C for 15 s and 60 °C for 4 min and final extension 60 °C for 15 s.

#### Parasite genotyping

*pfcrt* K76T and *pfmdr* N86Y mutations were assessed as described previously with minor modifications [31, 32]. Briefly, samples were prepared in a 5 µl reaction volume consisting of 2µl Lightscanner Master Mix (BioFire Diagnostics, Salt city, UT, U.S.A), 2.5 µl pre-amplified template, and 0.5 µl of primers and probes. The primers were in an asymmetric forward to reverse ratio of 1:5 (0.5 µM forward excess primer-, 0.1 µM reverse limiting primer, and 0.4 µM of the mismatched oligonucleotide at the end as a probe), and then amplified as follows amplification conditions 95 °C denaturation for 2 min, 50 cycles of 94 °C for 5 s and 66 °C for 30 s, and a pre-melt cycle of 5 s each at 95 °C and 37 °C. The change in fluorescence was recorded over a temperature range from 40 °C to 90°C on the Light Cycler 480 system (Roche Diagnostics, Applied Science, Germany) and analysed using the Call-It module within the Light Cycler software.

### Statistical analysis

Demographic and laboratory data of participants records were analysed using Stata version 13 (College Station, Texas, USA).

### Ethical clearance

Ethical clearance was obtained from the Regional Committee for Medical and Health Research Ethics (REC Western Norway) Ref no. 2016/1393/REK Vest and from the University of Zambia Biomedical Research Ethics Committee (UNZABREC) Ref no. 010-05-16. As this analysis was part of a larger study, ethical clearance for the larger study was also obtained from UNZABREC Ref no. 007-03-14. Permission to use data was obtained from the Ministry of Health. All data analysed were anonymized.

## Results

Of the 181 samples, 155 (85.6%) were successfully amplified. Table 1 shows the demographic characteristics of the study participants associated with these samples. There were 72 (46.5%) males and 83 (53.5%) females and the mean age was 20.8 years with a range from 1 year to 93 years.

**Table 1:** Demographic characteristics and K76T mutations

Characteristics	N	%	*Pfcrt* K76 (n)	Mixed (n)
Sex				
Male	70	46.4	1	1
Female	81	53.6		1
Age (years)				
< 5	25	16.6		
5–10	38	25.2		1
11–15	22	14.6		
16–25	22	14.6		
26–40	18	11.8	1	
41–94	26	17.2		1
Mean age (range)	20.8 (1–93 years)			
Province				
Southern	31	20.0	1	
Western	124	80.0		2

### Prevalence of *Pfcrt* K76T and *Pfmdr* N86Y

For *Pfcrt*, only 1 (0.6% 95% CI 0.02, 3.5) of the samples had the resistant allele with 2 (1.3% 95% CI 0.2, 4.6) samples having both resistant and sensitive alleles. Resistance, described, in this paper, as any sample containing a resistant alleles, was found in 3 samples [1.9% (95% CI 0.4, 0.6)]. For *Pfmdr* N86Y/F, all the 145 (100%) samples that successfully amplified had the sensitive allele (Table 2).

**Table 2:** Prevalence of *Pfcrt*76 and *Pfmdr* 86 mutations in Southern and Western Provinces

Codon	Resistant	Mixed	Sensitive
*Pfcrt* K76T (n = 155)	1 (0.6% 95% CI 0.02, 3.5)	2 (1.3% 95% CI 0.2, 4.5)	152 (98.1% 95% CI 94.4, 99.6)
*Pfmdr* N86F (n = 145)			145 (100% 95% CI 97.4, 101)

### Prevalence of *Pfcrt* K76T by province

**Fig. 1:**
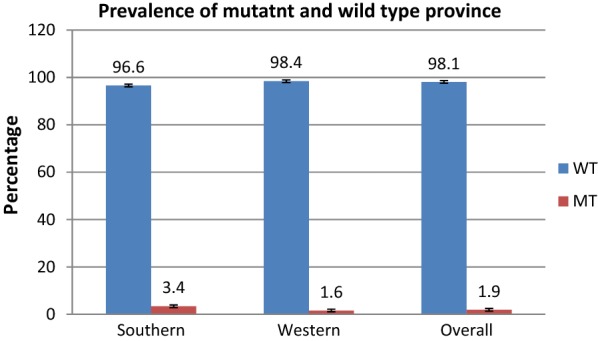
Prevalence of resistant and wild genes by Province

**Fig. 2:**
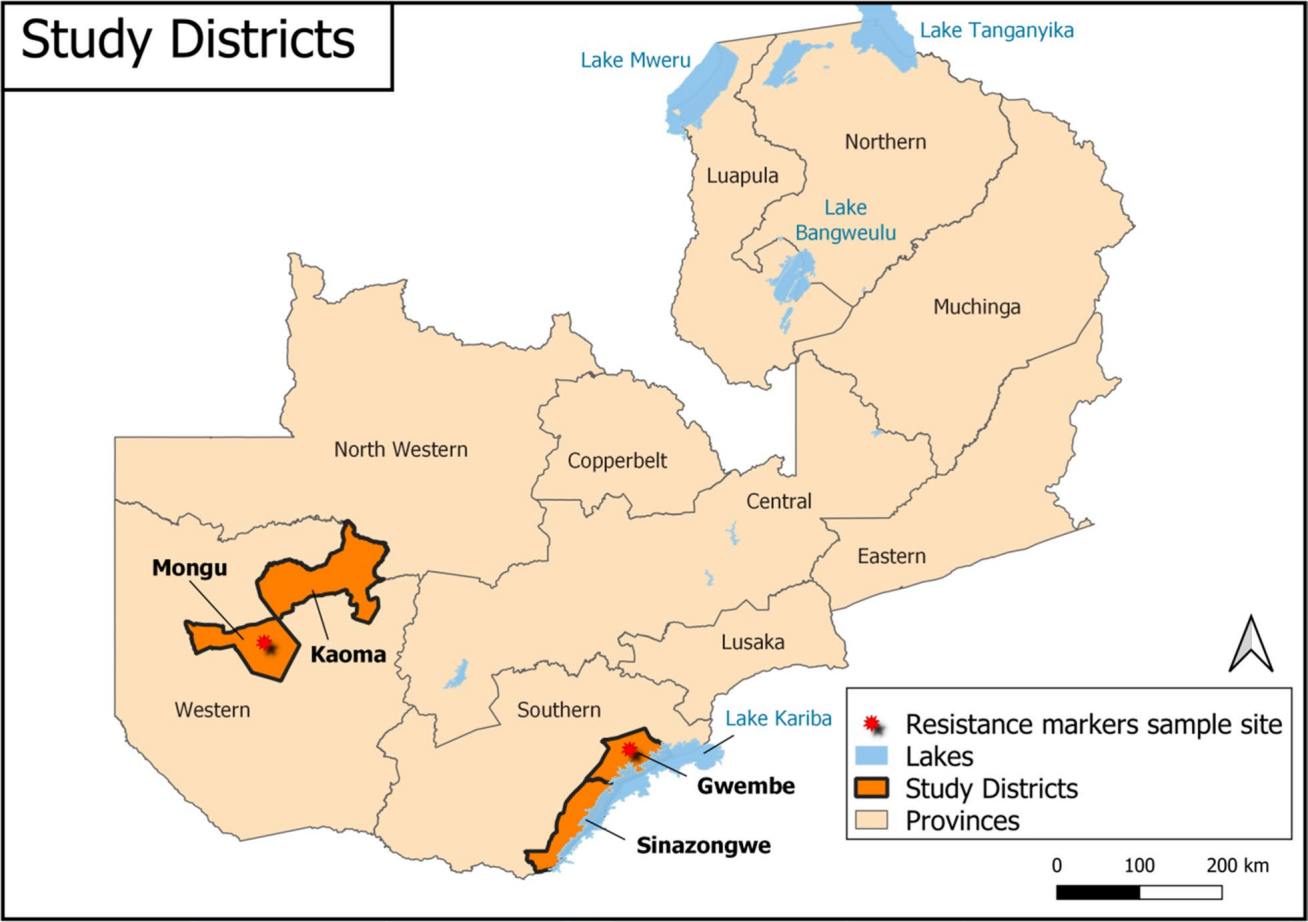
Study districts in Southern and Western Province

CQ resistance markers prevalence in Western Province was 2/122 [1.6% (0.2–5.7%)] while in Southern Province it was 1/29 [3.4% (0.08–17.2)] (Fig. 1). The sample from Southern Province only contained the resistant allele, while the two from Western Province were mixed, i.e. containing a copy of both sensitive and resistant alleles. Figure 2 shows the district where these samples carrying resistant markers came from.

## Discussion

Choloroquine (CQ) was a highly effective and cheap anti-malarial whose utility was drastically reduced through the evolution of anti-malarial drug resistance. This study reveals the return of CQ susceptible *P. falciparum* parasites in Southern and Western provinces of Zambia 14 years following the withdrawal of chloroquine as first line treatment in 2003. In this study the overall genotypic prevalence of resistant parasites was 1.9%, with no difference between Southern (3.4%) and Western (1.6%) provinces. A total of 3 infections (1.9%) contained the resistant genotype, however two of these samples were also carrying a sensitive allele, suggesting that this allele has almost reached fixation in the population. In contrast, there were no *Pfmdr N*86Y resistant alleles observed in the study. This mutation is a good indicator of treatment outcome as observed in a previous study where 68% of samples containing this mutation were associated with treatment failure [33].

**Fig. 3:**
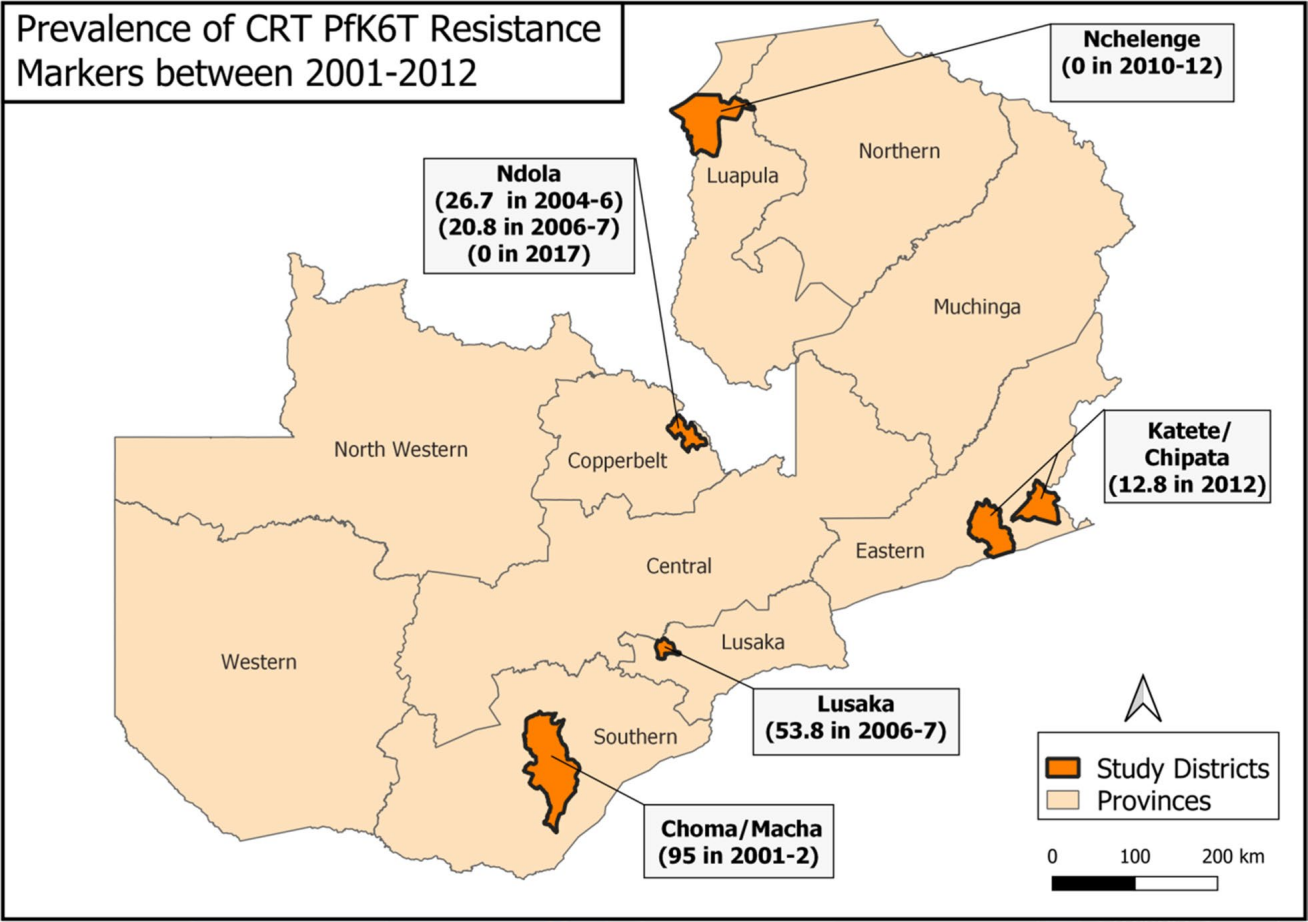
Pfcrt K76T resistance marker over the years from 2001–2012 in Zambia. Percentage of CQ resistant isolates in Zambia from 2001–2012, data from [21, 37–40]

These results mirror those observed in Northern Zambia, Malawi and Kenya [21, 34–36]. In Zambia, while not linear, there has been a general reduction in the prevalence of the chloroquine resistance genotypes over time. From a high of 95% resistance in Macha in 2001 (unpublished report as cited by Chileshe et al. [33]). This is seen from the gradual decrease observed from a multi-country study whose site in Zambia was from Ndola, Copperbelt province. It was conducted around 2004–2006 and 2006–2007 and revealed that resistance was around 20–30% [37]. On the other hand, around the same period, samples collected from Lusaka revealed 54% in 2006–2007 [38]. A further reduction to 14.8% was observed from samples collected in Katete and Chipata (unpublished data) and 0% in Nchelenge, Luapula Province, in 2012 [21]. Finally, 2017 samples from Ndola, Copperbelt province identified zero resistance markers [39] (Fig. 3).

The disappearance of CQ resistant parasites appears to be evolving at different rates in the region probably due to co-varying factors such as treatments given; malaria transmission intensity (the rate of recombination among the parasites); and the use of related drugs like Amodiaquine capable of maintaining drug pressure on *pfcrt* [40]. Amodiaquine has never been used in Zambia, but it has been the second-line treatment for malaria in neighbouring countries like Tanzania, whose nationals often travel to Zambia. As people travel from one place to another they import parasites [41]. In addition, infected individuals may carry resistant parasites; and these travellers could come with drugs from the neighbouring country that could alter the extent of drug pressure [42], these drugs could be shared with the Zambians they are visiting.

This re-emergence of chloroquine sensitive *P. falciparum* parasites, also called ‘chemo-reversion’, is interpreted as a result of the absence of chloroquine drug pressure leading to the rapid re-expansion of susceptible parasites that had survived in some malaria semi-immune individuals in the population during that period of chloroquine use [34]. Furthermore, it has been suggested that the large-scale use of lumefantrine may have accelerated chemo-reversion by placing a selective pressure towards wild type *Pfcrt* alleles [40].

This reduction of chloroquine resistant parasites could present a fresh chance to reintroduce chloroquine for malaria prevention especially in key vulnerable populations, as it is safe, well-tolerated in pregnancy at all stages and in children; and long-acting [21]. This could replace sulfadoxine-pyrimethamine and should be introduced as a combination therapy with artesunate or with other short acting drugs to extend the useful lifespan of each of the drugs [43]. In light of the development of drug resistance, a short-term periodical alternate use of combinations with chloroquine may be used to reduce the development of resistance.

### Study limitations

These results provide more information of the current state of *pfcrt*K76T allele in Western and Southern Provinces of Zambia which will be referred to when discussing anti-malarial resistance in Zambia. However, these results should be interpreted with caution: first, the samples size for the low transmission area was small making comparison difficult. Therefore, studies are required with a larger sample size that would provide more accurate estimates and comparisons of low and high transmission areas, especially after a long period of being low transmission areas in a place like Southern Province. Second, survey sampling in the two provinces was done differently making comparisons between the two more challenging. Finally, PCR bias cannot be ruled out completely as 26 samples did not amplify despite undergoing the pre-amplification process, this could have led to an under-or over-estimation of the prevalence.

## Conclusion and recommendation

This study suggests CQ sensitive alleles dominate the genetic landscape in Southern (low transmission) and Western (high transmission) Provinces of Zambia 14 years after the withdrawal of CQ. Routine national surveillance of molecular resistant markers for anti-malarial drugs should be included in the National Malaria Elimination Programmes as it strives for elimination. This surveillance should be countrywide and samples from patients visiting health facilities should be included. Armed with this data, the programme can better understand the genetic challenges, and opportunities, for delivering prompt, effective treatment and chemo-protection.

## Data Availability

The datasets generated and/or analysed during the current study are available from the corresponding author on reasonable request.

## Abbreviations

ACT: artemisinin-based combination therapy
AL: artemether–lumefantrine
Asn: asparagine
CQ: chloroquine
DBS: dried blood spot
DNA: deoxyribonucleic acid
ITNs: insecticide-treated nets
IRS: indoor residue spraying
LLITNs: long-lasting insecticide treated nets
Lys: lysine
LSM: larval source management
NPV: negative predictive value
NMEC: National Malaria Elimination Centre
NMCP: National Malaria Control Programme
MACEPA: Malaria Control and Elimination Partnership in Africa
MDA: mass drug administration
MIS: Malaria Indicator Survey
PCR: polymerase chain reaction
RDTs: rapid diagnostic tests
Thr: thyrosine
SMC: seasonal malaria chemotherapy
